# Role of the R349 Gene and Its Repeats in the MIMIVIRE Defense System

**DOI:** 10.3389/fmicb.2019.01147

**Published:** 2019-05-22

**Authors:** Said Mougari, Jonatas Abrahao, Graziele P. Oliveira, Jacques Y. Bou Khalil, Bernard La Scola

**Affiliations:** ^1^Unité MEPHI, Aix Marseille Université, IHU Institut Hospitalo-Universitaire Méditerranée Infection, Marseille, France; ^2^Laboratório de Vírus, Departamento de Microbiologia, Universidade Federal de Minas Gerais, Belo Horizonte, Brazil

**Keywords:** Mimivirus, virophage, MIMIVIRE, knock out, electronic microscopy

## Abstract

MIMIVIRE is a defense system described in lineage A Mimivirus (*Mimiviridae* family) that mediates resistance against Zamilon virophage. It is composed of putative helicase and nuclease associated with a gene of unknown function called R349, which contains four 15 bp repeats homologous to the virophage sequence. In a previous study, the silencing of such genes restored virophage susceptibility. Moreover, the CRISPR Cas-4 like activity of the nuclease has recently been characterized. In this study, a recently isolated Mimivirus of lineage A with R349 gene lacking 3 of 4 repeats was demonstrated to be susceptible to Zamilon. To reinforce the importance of the R349 gene in the MIMIVIRE system, we developed and presented, for the first time to our knowledge, a protocol for Mimivirus genomic editing. By knocking out R349 gene in a Mimivirus lineage A, we observed the replication of Zamilon, indicating that this gene is critical in the resistance against this specific group of virophages.

## Introduction

In recent years, several studies have shown that the endogenization of pathogen sequences to prevent their multiplication is a main mechanism of defense for living organisms. This phenomenon has been intensively discussed in the context of vertebrates evolution, where the number of integrated retroviruses reaches several thousand per organism (Colson et al., 2014). The current retrovirus epidemic in koalas is a good example of the endogenization process (integration and transmission to the progeny of the KoRV) that protects offspring from infection by these retroviruses (Tarlinton et al., 2006).

Bacteria and archaea have evolved several immune strategies to defend themselves against viruses, which are the most abundant biological entities in the biosphere (Suttle, 2005, 2007; Rohwer and Thurber, 2009; Horvath and Barrangou, 2010). These immune strategies have been classified into innate and adaptive defense systems (Koonin et al., 2016). The innate immunity systems are represented by diverse types of restriction modification (RM) systems, while the CRISPR (clustered regularly interspaced short palindromic repeats)-Cas (CRISPR-associated genes) system is the unique adaptive immune mechanism that mediates defense against viruses (Orlowski and Bujnicki, 2008; Karginov and Hannon, 2010).

The CRISPR-Cas defense system involves the excision of DNA fragments from alien sequences and their integration into CRISPR arrays (Sorek et al., 2008; Koonin and Makarova, 2009; van der Oost et al., 2009; Deveau et al., 2010; Horvath and Barrangou, 2010; Karginov and Hannon, 2010; Makarova et al., 2011). This mechanism is known as the adaptation and requires the intervention of the Cas machinery. The integrated sequences (spacers) create an immune memory capable of protecting the host cell during new encounters with the invading agents. Indeed, the processed transcripts of the spacers are used as guides for the Cas nucleases (effector) to recognize and cleave the target genome. This immune system is highly specific and protects diverse bacterial organisms from a wide variety of invading nucleic acids like phages. The latter are involved in a permanent arms race with their prey by developing various immune evasion strategies. Perhaps the most astonishing strategy is to counterattack an innate bacterial defense mechanism by encoding a complete CRISPR-Cas response (Seed et al., 2013).

The discovery of giant viruses of amoeba has challenged our definition of what a virus is (La Scola et al., 2003; Colson et al., 2018). In addition to the giant size of their capsids and genomes, giant viruses of the family *Mimiviridae* have been shown to be, themselves, the prey of other viruses, thus highlighting a prominent turn of events in the virology field (Raoult et al., 2004; La Scola et al., 2008; Colson et al., 2017). The newly discovered viruses of giant viruses were named virophages because of their functional analogy with bacteriophages. Virophages parasitize the viral factory of giant viruses supposedly by hijacking the transcription and replication apparatus of their host virus to express and replicate their own genomes (Claverie and Abergel, 2009). Sputnik is the first isolated virophage and was able to infect the three phylogenetic groups that cluster in the *Mimiviridae* family (group A, B, and C) (Gaia et al., 2013). In contrast, Zamilon, the second isolated virophage, was able to replicate with mimiviruses from group B and C, but not with those belonging to group A (Gaia et al., 2014).

The selective resistance of Mimivirus group A to Zamilon virophage led us to look for integrated sequences of virophages that we described as “MIMIVIRE” for Mimivirus virophage resistance element. Different from the CRISPR to which it has been compared by analogy, the MIMIVIRE operon contains a gene named R349 with 4 small repeats of the virophage targets (Levasseur et al., 2016). The silencing of 3 genes from the MIMIVIRE operon (encoding a helicase-like gene, a nuclease-like gene and the gene containing repeats) abolished the MIMIVIRE activity, a phenomenon that we have characterized as the result of an adaptive defense system (Levasseur et al., 2016). This hypothesis has been controversial in the literature (Claverie and Abergel, 2016). However, nuclease and Mimivirus helicases have already been expressed to identify their role, and a new recent study reported, after expression and crystallization, that the nuclease has a Cas-4 activity (Bekliz et al., 2016; Dou et al., 2018). On the other hand, the molecular bases of the interference mechanism mediated by the MIMIVIRE system remain enigmatic, notably the role of the repetitive sequences.

We have recently isolated and sequenced a new Mimivirus lineage A strain from a Human sample (Moal et al., 2018). Here, we analyzed its genome and identified a divergent MIMIVIRE operon. Indeed, the R349 gene in this virus does not present the repeated pattern observed in the other mimiviruses of lineage A. We then investigated the functional consequence of the absence of the repetitive motif by testing the sensibility of this new Mimivirus strain to Zamilon virophage. To reinforce the importance of the R349 gene in the MIMIVIRE system, we developed and presented, for the first time to our knowledge, a protocol for Mimivirus genomic editing. By knocking out R349 gene in Acanthamoeba Polyphaga Mimivirus (APMV), we observed the replication of Zamilon, indicating that this gene is critical for the resistance against this specific group of virophages.

## Materials and Methods

### Mimivirus Strain U306 and Zamilon Virophage Production

#### Mimivirus Strain U306 Production and Genome Analysis

*Acanthamoeba castellanii* trophozoites at a concentration of 5.10^5^ cell/ml in PYG (Peptone yeast extract glucose) medium were used to produce Mimivirus U306 and APMV (La Scola et al., 2003; Moal et al., 2018). A Multiplicity of infection (M.O.I.) of 10 TCID_50_ (median Tissue Culture Infectious Dose) was used to infect the host cells with each virus strain. After 48 h of incubation at 32°C, the virus suspension was centrifuged at 1000 g for 10 min. The supernatant containing the virus progeny was then filtered across a 0.8 μm membrane to remove residual amoebas and cysts. For a second time, the virus suspension was washed three times with Page’s modified Neff’s amoeba saline (PAS) medium by high-speed centrifugation (10,000 g for 10 min) to pellet the virus particles.

The genome of Mimivirus U306 was previously submitted to the EMBL-EBI database under accession number LT717347. Here, GeneMarkS was used for the prediction of coding DNA sequences. The R349 homologs in Mimivirus U306 were found using BLASTn searches (Basic Local Alignment Search Tool).

#### Zamilon Virophage Production

Megavirus Courdo 11 was used to produce Zamilon virophage by co-culture in *A. castellanii* within PYG medium (La Scola et al., 2010). The co-culture was incubated at 32°C until complete lysis of cells. The virus-virophage suspension was centrifuged at 10,000 g for 10 min and successively filtered through 0.8, 0.45, and 0.22 μm pore filter to remove giant virus particles and residual amoebas. The Zamilon particles were concentrated by ultracentrifugation (60,000 g for 2 h) and the pellet was then resuspended with PAS medium. In the last step, the highly concentrated virophage suspension was submitted to a final round of ultracentrifugation (60,000 g for 2 h) across a 15% sucrose layer to obtain a pure Zamilon pellet, which was resuspended in 1 ml PAS medium and stored at –80°C. The absence of giant virus particles was confirmed by negative staining electron microscopy.

### APMV R349-KO Viruses

#### Plasmid Construction for Knock-Out Experiments

The R349 gene in APMV was knocked out by homologous recombination method in amoebas infected with APMV and transfected with the recombination vector. The backbone of this vector was the PLW44 plasmid (kindly provided by Dr. Flávio da Fonseca, Universidade Federal de Minas Gerais), which contains enhanced green fluorescent protein (eGFP) gene as reporter, under the control of an AT-rich promoter region, similar to the promoter motif predicted for mimiviruses (Claverie, 2005; Abrahão et al., 2018). We inserted both the upstream and downstream regions of mimivirus R349 gene into PLW44, flanking the eGFP gene and its promoter. The APMV R349 flanking regions (500 bp each) were separately amplified by PCR and sub-cloned into pGEMT-easy vector (Promega, United States). The details of the primers used are listed in Table 1. These plasmids were propagated in *Escherichia coli* DH5alpha and were then extracted by using Minipreps plasmid extraction kit (Promega, United States). The R349 flanking regions were released from pGEMT-easy vector after digestion with EcoRI (flanking region 1) and SmaI/PstI (flanking region 2) (Promega, United States). In parallel, the vector PLW44 was digested with the same enzymes and the fragments were then inserted (T4 DNA ligase, Promega, United States). After one round of PLW44-eGFP-Flank1/2 propagation in *Escherichia coli* DH5alpha, the vector was purified as previously described, quantified in nanodrop1000 spectrophotometer (Thermo Fisher Scientific, United States) and stored at −80°C until use.

**TABLE 1:** Primers used for R349 flanking regions cloning into PLW44 vector.

**Primers**	**Sequences^a^**
FwPLW F1 EcoRI	5′AACTCTTA**GAATTC** GGATATTTTGAATTGTCTGTTCA3′
RvPLW F1 EcoRI	5′TTATGTA**GAATTC** TTTGGAAGGTGAAATTTATATGTA3′
FwPLW F2 SmaI	5′CAACAAT**CCCGGG** TCCACTAAATTAATCTCCGATATT3′
RvPLW F2 PstI	5′AAGATCT**CTGCAG** CATGGATCATTATGTAGTGACGCT3′

*^a^Restriction sites are underlined.*

#### Transfection and Virus-Vector Recombination

Prior to transfection, fresh *Acanthamoeba castellanii* cells (ATCC 30010) were washed twice with peptone-yeast-glucose (PYG) medium, one million cells were centrifuged at 1200 g for 10 min and the media was removed. Cells were resuspended in 2 ml PYG medium and placed in a six-well culture plate (Thermo Fisher Scientific, United States). DNA was prepared for transfection as follows: one microgram of PLW44-eGFP-Flank1/2 plasmid was incubated with 15 μl Lipofectamine 3000 reagent (Thermo Fisher Scientific, United States) and PYG medium, according to the manufacturer’s instructions. 3 × 10^6^ TCID_50_ APMV was added, and the solution was mixed gently by pipetting up and down. The mixture was added to the cells in a six-well culture plate. The cells were then incubated at 32°C, and after complete cell lysis (after 48 h approximatively) the extract containing recombinant (KO R349) and wild-type (WT) viruses was collected and stored at −80°C.

#### APMV R349-KO Viruses Screening

First, specific PCR systems targeting the recombinant virus were designed and performed on the DNA extracted from the previous step, in order to confirm the insertion of the eGFP gene instead the R349 gene (Table 2). One million of fresh *A. castellanii* was then infected in a 25 cm^2^ flask with 1 ml of extract of viruses obtained in the transfection experiment (containing both R349-KO and wild type populations). After 30 min of adsorption, the inoculum was removed and 5 ml of Page’s modified Neff’s amoeba saline (PAS) was added to the flask. The infected cells were incubated for 8 h at 32°C, and were then detached from the flask, centrifuged for 10 min, 1200 g, and the pellet was resuspended in 500 μl of PAS buffer. This time point represents a late phase of the Mimivirus cycle where virion morphogenesis has already begun and fully assembled viral progeny have already been observed in Mimivirus infected cells (Suzan-Monti et al., 2007; Andrade et al., 2017). In parallel, the same procedure was performed using APMV wild-type infected amoebas. The infected cells were then submitted to BD FACS Scan flow cytometer (BD, United States) for recombinant viruses screening and sorting. We sorted between 1,000 and 2,000 cells. The eGFP fluorescence cutoff was defined based on uninfected and APMV wild-type infected cells. The amoebas presenting a level of fluorescence higher than the defined-cut off were selected, sorted and collected in PAS solution (Supplementary Figure S1). These cells were transferred to a 25 cm^2^ flask containing one million of fresh amoebas and incubated for 48 h at 32°C.

**TABLE 2:** Primers used for KO-R349 recombinant viruses screening and sequencing.

**Primers**	**Sequences**
Fw-KO-Up	5′CTTGTTGAATTGGGTCTTGGTC3′
Rv-KO-Up	5′GAACAGCTCCTCGCCCTTG3′
Fw-KO-Dw	5′AGATCCGCCACAACATCGAG3′
Rv-KO-Dw	5′CTTGGATCACGTCCCAACCA3′
Fw-R349-screening	5′GGATGTCGTAATTGTGCCTGA3′
Rv-R349-Screening	5′AGGCCCATCACCAAAACCAA3′
Fw-GFP-screening	5′GGCAAGCTGACCCTGAAGTT3′
Rv-GFP-screening	5′CTTGTAGTTGCCGTCGTCCT3′

The lysate containing the R349 KO recombinant viruses was collected and submitted to five more rounds of clonal purification. To this end, 500,000 *A. castellanii* were infected in 25 cm^2^ flask with the lysate produced during the last step (MOI of 1 TCID_50_). After 30 min, the inoculum was removed, the cells were centrifuged for 10 min at 1200 g and the pellet was resuspended in 100 μl. These cells were then inoculated onto a non-nutrient agar plate, covered by fresh heat-inactivated *E. coli* DH5-alpha (Invitrogen, United States). The inoculation was performed equidistantly by dropping nine 10 μl droplets of the resuspended pellet. The plate was then incubated for 8 h at 32°C. At this point, it was possible to observe the amoeba’s movement onto the agar surface. Under an optical microscope, isolated amoebas were marked with a pen (without opening the plate) and were then collected individually with a small tip or needle under sterile conditions. Each collected amoeba was transferred to a well containing 40,000 fresh amoebas in a 96 well plate (200 μl of PAS buffer per well). After 24 h of incubation at 30°C, the wells were observed under a microscope to check for cytopathic effects (CPE). In wells where the CPE was observed, 30 μl aliquots of each viral suspension were collected and submitted to DNA extraction using the EZ1 DNA Tissue Kit (Qiagen, Germany) according to the manufacturer’s instructions. Screening PCRs targeting R349 or eGFP gene were then performed to confirm the recombination and the lack of contamination with the wild-type (Table 2), the products were visualized on an agarose 1% gel using SYBR safe buffer (Invitrogen, United States). The remaining 170 μl were used for four new rounds of recombinant viruses screening and purification. After the final round of KO virus purification, the remaining 170 μl containing only KO virus were transferred to a 25 cm^2^ flask with 500,000 of fresh amoebas and incubated for 48 h at 32°C to propagate the KO virus. After lysis, a small background of WT viruses was detected by qPCR. This probably indicate that a very small amount of WT virus was present in the last cycle of the purification process but was not detected by the R349-PCR system due to its low concentration. This production containing a mixture of KO and WT viruses was used to perform Mimivirus-virophage permissiveness experiments. A MOI of 1 TCID50 of KO virus was used for the virophage permissiveness assay to reduce the WT contamination and avoid the co-infection WT-KO virus. We are currently working to improve our purification system, by adding selection factors to genetic constructions, such as geneticin (ongoing).

Regarding the construction of the APMV R349-revertant virus, the same procedures were performed. However, the lack of eGFP and the presence of R349 were the selected screening factors, by using fluorescence detection and PCR (Table 2). The plasmid used for R349-revertant construction was rescued after the homologous recombination of PLW44-eGFP-Flank1/2 and APMV. An aliquot of this cell extract was submitted to DNA extraction and electrophoresis, and the band corresponding to the plasmid was then recovered from the 1% agarose gel (Qiagen gel extraction kit), transformed in *E. coli* DH5alpha, propagated and purified as previously described. This plasmid was used to re-insert the R349 gene into the genome of KO R349 Mimivirus by homologous recombination, as described above. All plasmids and recombinant (viral) regions described in this work were sequenced 4 times to confirm the absence of mutations in the related ORFs, rescue markers and flanking regions.

**FIGURE 1:**
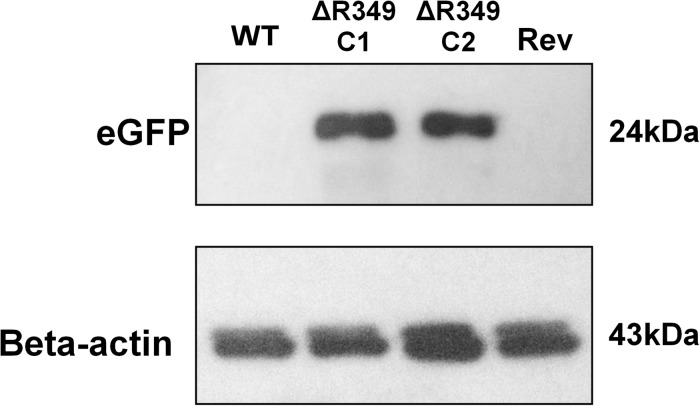
eGFP and amoebal beta-actin detection by western blot. *A. castellanii* cells were infected with APMV-Wild-type, APMV-R349-KO (two different clones) and APMV R349-revertant at M.O.I. of 10 TCID_50_. The cells were collected 8 h post-infection, submitted to protein extraction and then to western-blot targeting eGFP and amoebal beta-actin. The figure shows that, as expected, APMV-R349-KO clones express eGFP.

To check the presence/absence of eGFP protein in the extract of cells infected (8 h post-infection) with APMV-R349-KO, APMV R349-revertant or APMV-Wild-type, western-blot assays were performed using anti eGFP and anti-Beta-actin antibodies (F56-6A1.2.3 and AC-15 ab6276 – Abcam) (Figure 1).

### Zamilon Infection of APMV, APMV-R349-KO, APMV R349-Revertant, and Mimivirus U306

To check whether Mimivirus U306, APMV R349-KO, or APMV R349-revertant are permissive to Zamilon replication, the infections were prepared as described by Levasseur et al. (2016). The experiment was carried out three times independently, in duplicate. DNA extraction and PCRs were performed as described by Levasseur et al. (2016) as well and analyzed by ΔCt method considering times 0 and 24 h post-infection. The results were then expressed in Relative quantification (quantification of the increase in Zamilon DNA concentration at H24, relative to its concentration at H0). Transmission electron microscopy experiments were conducted to assess Zamilon growth observed by qPCR with Mimivirus U306. Co-infected cells were washed three times with phosphate-buffered saline (PBS) solution and fixed overnight with 2% glutaraldehyde in 0.1 M cacodylate buffer. Cell pellet was then washed three times with 0.1 M cacodylate buffer, and subsequently fixed for 1 h with 1% osmium tetroxide in 0.1 M potassium ferricyanide solution. After three successive washes in distilled water, samples dehydration was performed in increasing ethanol concentrations (30, 50, 70, 96, and 100%) before embedding in Epon 812 resin. Sections (70 nm) were finally post-stained with 3.5% uranyl acetate and lead citrate before examination with a transmission electron microscope.

## Results

In a previous large-scale survey, aimed at reporting the presence of a giant virus in the urine of 480 kidney transplant recipients, we reported the isolation of a new member of the *Mimiviridae* family (Moal et al., 2018). The phylogenetic tree based on DNA-dependent RNA polymerase gene has classified this new Mimivirus (Mimivirus U306) in the lineage A of the *Mimiviridae* family (Moal et al., 2018).

The genome of Mimivirus U306 available in the EMBL-EBI database under accession number LT717347 has now been analyzed. Coding DNA sequences were predicted using GeneMarkS. We found that Mimivirus U306 genome contains 936 predicted genes.

**FIGURE 2:**
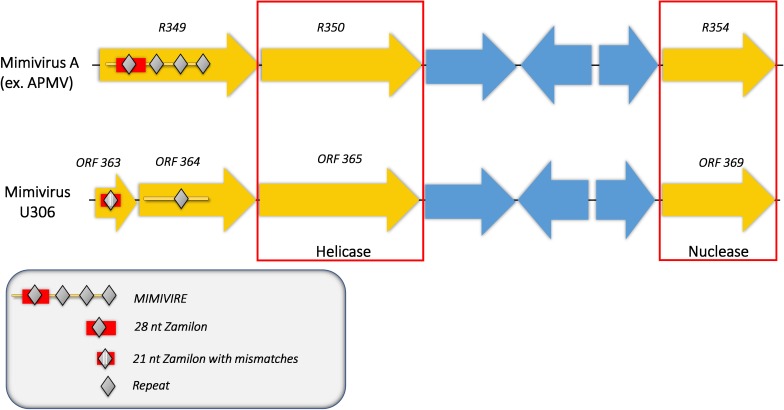
Comparison of the MIMIVIRE sequences between Mimivirus U306 and APMV. Mimivirus U306 lacks the repetitive motifs and presents a truncated MIMIVIRE sequence compared to APMV and the other Mimiviruses lineage A. Mutations are indicated by white lines.

**FIGURE 3:**
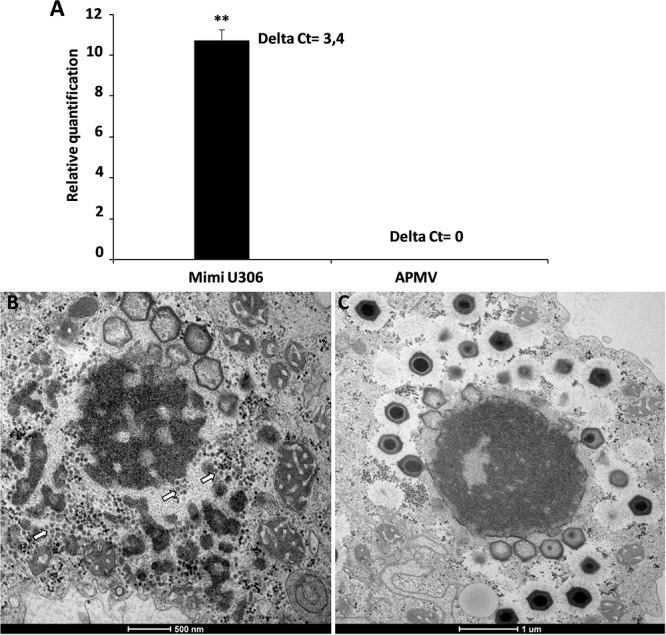
The replication of Zamilon in *Acanthamoeba castellanii* co-infected with Mimivirus U306 or APMV. **(A)** Graph depicting the replication of the virophage with Mimivirus U306 or APMV measured after 24 h by qPCR. Delta Ct corresponds to the difference between Ct value specific to virophage at H0 and H24. **(B,C)** Transmission electron microscopy images after 16 h of Zamilon infection. **(B)** Zamilon was able to infect and replicate within the viral factories of Mimivirus U306 (arrows). **(C)** No virophage progeny was observed in the viral factories of APMV. ^∗∗^Student’s *t*-test, *p* < 0.005.

We then analyzed the Mimivirus U306 genes for the presence of a MIMIVIRE system hallmark, a common feature found in all mimiviruses genomes from lineage A described so far. The ortholog of R349 in Mimivirus U306 consists of two different genes, respectively, ORF 363 and ORF 364. We identified a truncated copy of the MIMIVIRE system sequence, scattered between these genes (Figure 2 and Supplementary Table S1). Indeed, the ORF 363 gene contains a 21-nucleotide-long sequence of Zamilon instead of the 28 nucleotide sequence found in the classical MIMIVIRE system (Levasseur et al., 2016). We found that this 21-nucleotide-long stretch presents 3 mismatches compared to the original 28 nucleotide sequence located in open reading frame 4 (ORF 4) of the Zamilon genome and the R349 of the other mimiviruses from the group A (Figure 2 and Supplementary Figure S2). Moreover, the repetitive motifs are lost in the Mimivirus U306 MIMIVIRE copy as the ORF 364 gene only contains a single copy of the 15-nucleotide sequence that was identical to Zamilon, versus four repeated copies described in the MIMIVIRE of the other mimiviruses lineage A (Figure 2). This single 15 nucleotide repeat perfectly matched the four repeated sequences (and thus the initial 28 nucleotide sequence) found in the R349 of APMV and the other mimiviruses A (Supplementary Figure S2). Using the quantitative PCR, we observed for the first time the permissiveness of a lineage A Mimivirus to Zamilon with an increase in the DNA concentration of the Zamilon virophage after 24 h post-infection and observation of Zamilon particles infecting the viral factory of the Mimivirus (Figure 3). In addition, real-time PCR and transmission electronic microscopy confirmed the results of previous studies showing that Zamilon is not able to replicate in mimiviruses from lineage A including APMV (Figure 3).

The second aim of this work was to reinforce the importance of the R349 gene in the resistance against Zamilon. Indeed, although its implication has been validated in the initial MIMIVIRE study by silencing experiment with small interfering RNAs (siRNAs) (Levasseur et al., 2016), one might suppose that the permissiveness of Mimivirus A to Zamilon has been induced by the knock-down of an unintended target within amoeba or Mimivirus. We therefore first confirmed the absence of sequences identical to those silenced in the amoeba by similarity searches.

**FIGURE 4:**
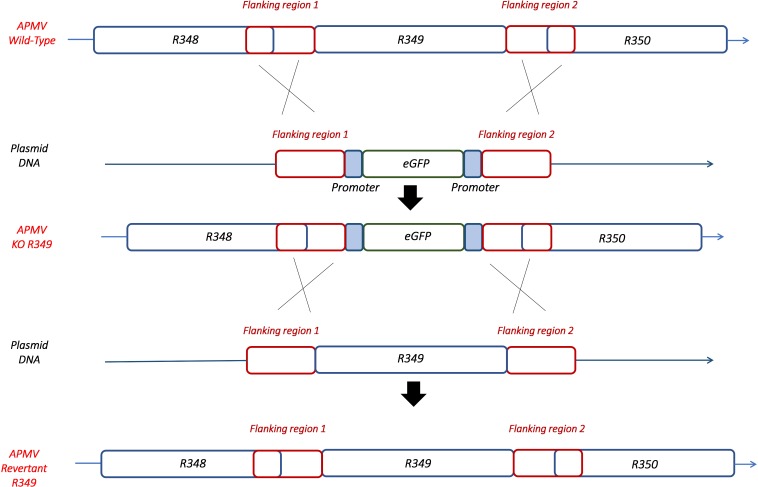
Homologous recombination strategy used to create APMV KO-R349 and APMV R349 revertant. The R349 gene was replaced by the eGFP marker derived by the AT-rich promoter to generate the APMV KO-R349 mutant. APMV R349 revertant was created by re-inserting the R349 gene in APMV KO-R349 instead of the eGFP sequence.

**FIGURE 5:**
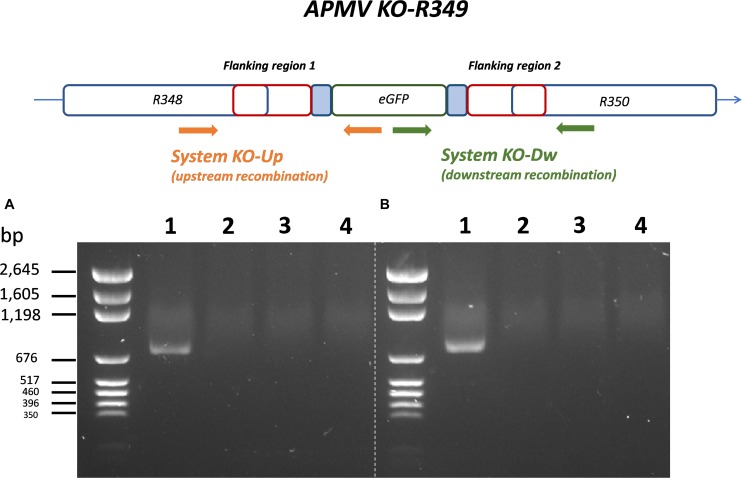
Specific PCR characterization of the recombinant KO-R349 obtained from the transfection experiment. Two specific PCR systems were used to confirm the recombination and the insertion of eGFP instead of the R349 gene in the KO-R349 mutant. The first system, KO-Up, targets recombination upstream of the R349 gene **(A)**, while the second system, KO-Dw, targets recombination downstream of the target gene **(B)**. (1) DNA extracted from the lysate obtained in the transfection experiment, (2) DNA extracted from *Acanthamoeba castellanii* cells infected with the APMV wild type, (3) PLW44-eGFP-Flank1/2 plasmid was used as template for the PCR, (4) negative control (nuclease-free water).

By knocking-out R349 gene from the APMV genome, we were able to confirm and highlight the central role of the R349 gene in the defense mechanism against Zamilon. We have created a Mimivirus deletion mutant lacking the R349 gene by homologous recombination, in which the enhanced green fluorescent protein gene has been inserted to replace the Mimivirus R349 gene (Figure 4). The insertion of eGFP marker in the appropriate locus, instead of the R349 gene, was confirmed by using 2 specific PCR systems (Figure 5). The first primer pair, Fw-KO-Up and Rv-KO-Up, were designed to amplify the recombination product upstream of the eGFP. In this system, Fw-KO-Up targets the R348 gene (outside the flanking region1), while Rv-KO-Up targets the eGFP (Figure 5). On the other side, Fw-KO-Dw and Rv-KO-Dw were designed to specifically target a sequence in the eGFP and a region in the R350 gene downstream of the flanking region 2, respectively (Figure 5). The product of each system was sequenced and confirmed the success of the recombination (The primers used for the PCR and sequencing experiments are listed in Table 2).

**FIGURE 6:**
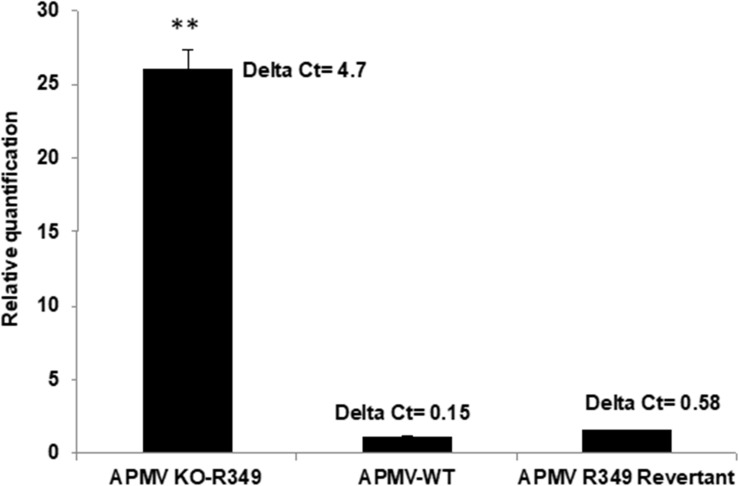
Graph depicting the replication of Zamilon DNA in *A. castellanii* co-infected with APMV KO-R349, APMV-WT, or APMV R349 revertant. The replication of each virophage was measured after 24 h using qPCR. Delta Ct corresponds to the difference between Ct value specific to virophage at H0 and H24. ^∗∗^Student’s *t*-test, *p* < 0.005.

After rounds of screening and sorting by flow cytometry and PCR targeting recombinant viral clones (Supplementary Figure S1), the KO R349 Mimivirus was submitted to assays to check its permissiveness to Zamilon virophage in *Acanthamoeba*, as described by Levasseur et al. (2016). In addition, APMV-wild type and a revertant virus (in which the R349 gene was re-inserted instead of eGFP as shown in Figure 4) were assayed. After 24 h post-infection, we observed a ∼25-fold increase in the Zamilon genome load in cells co-infected with the R349-KO virus (Figure 6). In contrast, we could not observe Zamilon genome replication in cells co-infected with APMV-wild type, nor in cells co-infected with the revertant virus. For the latter, a slight increase in Zamilon DNA concentration was observed compared to the APMV-wild type (less than 0.5 PCR cycle difference between revertant APMV and APMV-wild type) (Figure 6). Such a difference probably does not seem significant and therefore unreliable to draw a strong conclusion. Otherwise, one might suppose that this increase could be explained by the presence of a small background of KO-R349 virus in the revertant Mimivirus production, which allowed the Zamilon virophage to replicate. These results were reinforced by negative staining microscopy, in which a substantial amount of virophage particles were observed only in cells co-infected with the R349-KO virus (data not shown).

## Discussion

In this study, we presented evidences of the role of the repetitive sequences in the resistance mechanism mediated by the MIMIVIRE system against Zamilon virophage. We demonstrated that the new Mimivirus U306, which has a MIMIVIRE sequence containing a single repeat instead of 4 virophage repeats, is permissive to Zamilon infection. This finding is evocative to what has been observed for the bacterial CRISPR-Cas system. Indeed, it has been shown that the addition of multiple spacers targeting the same phage sequence in the CRISPR-array of *Streptococcus thermophilus* can increase the level of its resistance to the phage (Barrangou et al., 2007). We speculate that the number of four repeats in MIMIVIRE increases the probability that the repeat transcript identifies the target sequence and thus triggers the interference mechanism. However, further experiments are required to validate this hypothesis. In addition, it has also been reported that *S. thermophilus* mutant, which contains a single repeat rather than the repeated consensus in the CRISPR-array, was also sensitive to the phage despite the presence of spacers targeting it (Barrangou et al., 2007). These results, along with our observations, probably indicate that the interference mechanism of both MIMIVIRE and CRISPR-Cas systems require that the target sequence (spacer for CRISPR and repeat for MIMIVIRE) should be inserted into a specific genetic context. This genetic context may be the succession of a few target repeats for MIMIVIRE, versus alternation repeat-spacer in CRISPR. On the other hand, the 28-nucleotides Zamilon sequence found in the classic MIMIVIRE consists in Mimivirus U306 of only 21-nucleotides sequence presenting several mutations compared to the target in Zamilon. In some CRISPR-Cas system types, a perfect match is required between the spacer and the protospacer for target recognition and resistance. A single polymorphism between the spacer and the target sequence has been shown to be sufficient to allow the phage to infect the CRISPR-immunized bacterial host (Barrangou et al., 2007; Deveau et al., 2008). Therefore, the presence of mutations could be one of the causes of permissiveness to Zamilon, or the fact that the MIMIVIRE sequence is truncated in between two genes in the U306 Mimivirus genome. Recently, an alternative scenario to the adaptive immune system has been proposed to explain the mechanism of resistance of Mimivirus lineage A to Zamilon (Claverie and Abergel, 2016). The process of Zamilon inhibition has been interpreted by a protein interference, mediated by a restriction factor analog, which is encoded by the R349 gene. According to this model, this restriction factor-like protein is supposed to use the repeat motifs to interfere with the action of its target in Zamilon virophage. Although experimental demonstrations are required to verify this hypothesis, the permissiveness of Mimivirus U306 to Zamilon would not question it. Furthermore, as described above, the homolog of R349 gene in Mimivirus U306 presents several mutations that cut it into two separated genes. Therefore, this gene is no longer functional and therefore no longer produces the putative restriction factor-like required for the defense against Zamilon infection. One might suppose that this alleviate the protein-mediated inhibition and thus allows Zamilon to replicate in Mimivirus U306. In addition, we screened here the R349 orthologous proteins in Mimivirus U306 for the presence of the peptidic motif “Asp-Asn-Glu-Ser” (DNES), that has previously been predicted to be implicated in the protein-based interference mechanism (Claverie and Abergel, 2016). Interestingly, we identified only one copy of the “DNES” motif in Mimivirus U306 (located in the ORF 364 gene) instead of six copies found in the R349 gene of APMV. On the other hand, ORF4 of Zamilon (putative target of MIMIVIRE) has been shown to contain two copies of DNES motif (Claverie and Abergel, 2016), which is higher than what is found in Mimivirus U306. This finding raises probably an alternative scenario in which the occurrence of mutations in the R349 homolog of Mimivirus U306 causes this gene product to lose the repeated peptidic motif implicated in the antagonist activity against the ORF4 product of Zamilon. This probably allows the ORF4 protein to trigger the proteins-interactions necessary for the expression and the replication of Zamilon virophage.

Afterward, we performed homologous recombination to knock-out the R349 gene of Mimivirus by replacing it with the enhanced green fluorescence protein (eGFP) gene. This latter was used during the process of selection and purification of the recombinant clone. At the end of the fluorescence-based selection process, we obtained a recombinant Mimivirus lacking the R349 gene. Our results confirm that the R349 gene has a critical role in the resistance of Mimivirus lineage A to Zamilon, since this latter was able to infect and replicate with R349-deficient Mimivirus. This observation was strongly confirmed by generating the R349-revertant Mimivirus that was resistant to Zamilon by knocking-in the R349 gene in the genome of the R349 deficient strain (in the same locus). Unfortunately, since selection is made on fluorescent virus factories, not on single fluorescent virus particles, and in the absence of a pressure selection system, it was not possible to obtain stable R349-KO viruses. Nevertheless, this first transformation of a giant virus is encouraging. In the future, we will have to look for a selection system that allows us to obtain stable transformed viruses.

## Conclusion

In conclusion, we demonstrated herein that the R349 gene contained within the MIMIVIRE system is crucial for the protection of the Mimivirus lineage A from Zamilon infection. We additionally highlighted the functional role of the integrity of the R349 in the process of resistance to Zamilon by demonstrating that mimiviruses that lack the repetitive consensus and present mutations in this gene are susceptible to virophage. In the future, it would be an obvious option to use the protocol of genome editing described here to generate some derivative constructs, like mimiviruses containing partial R349 sequences. This would allow to determine precisely which element in the R349 gene is relevant in conferring the resistance (perfect match to the Zamilon sequence and/or number of repetitions). Another interesting point that could be investigated in the future is the effect of Zamilon on the replication cycle of Mimivirus U306 and the KO-R349 virus. Indeed, although this virophage was not able to affect mimiviruses from lineage B and C, its impact on mimiviruses lineage A has never been explored (Since it was not able to infect any virus from this lineage). We believe that studying its involvement would give insight to understand why only mimiviruses from lineage A have evolved a defense system against this virophage.

These elements reinforce the MIMIVIRE system and make it possible to propose that giant viruses, like other organisms, be involved in an arms race to survive and multiply despite parasitism, including that of the virophage.

## Author Contributions

SM performed the experiments, analyzed the results, and wrote the manuscript. GPO performed the experimentations. JB performed electronic microscopy experiments and analysis. JA and BL conceived the study and wrote the manuscript.

## Conflict of Interest Statement

The authors declare that the research was conducted in the absence of any commercial or financial relationships that could be construed as a potential conflict of interest.

## Supplementary Material

FIGURE S1 APMV R349-KO infected amoebas screening and sorting by BD FACS Scan flow cytometer. The cutoff was defined based on the background fluorescence detected in uninfected and APMV-wild-type infected cells.

FIGURE S2 Sequence homology representation between different Zamilon sequences present in the MIMIVIRE of APMV and U306 strain. The 28-nucleotides sequence of Zamilon present in the initial R349 gene of mimiviruses A is shortened to a sequence of 21 nucleotides with 3 mismatches (in red) in the ORF 363 of Mimivirus U306 strain. The repeat of 15 nucleotides (albeit single) located in the ORF 364 match perfectly the original 28-nucleotides sequence (and thus the four repeated sequence) found in the other mimiviruses from lineage A.

TABLE S1 Nucleotide and protein sequences of the Mimivirus U306 MIMIVIRE copy and their annotation.
