# Supplementary figures and images for: Role of the R349 Gene and Its Repeats in the MIMIVIRE Defense System
Source: Front Microbiol. 2019 May 22;10:1147. doi: 10.3389/fmicb.2019.01147 (PMC6538805; doi:10.3389/fmicb.2019.01147)

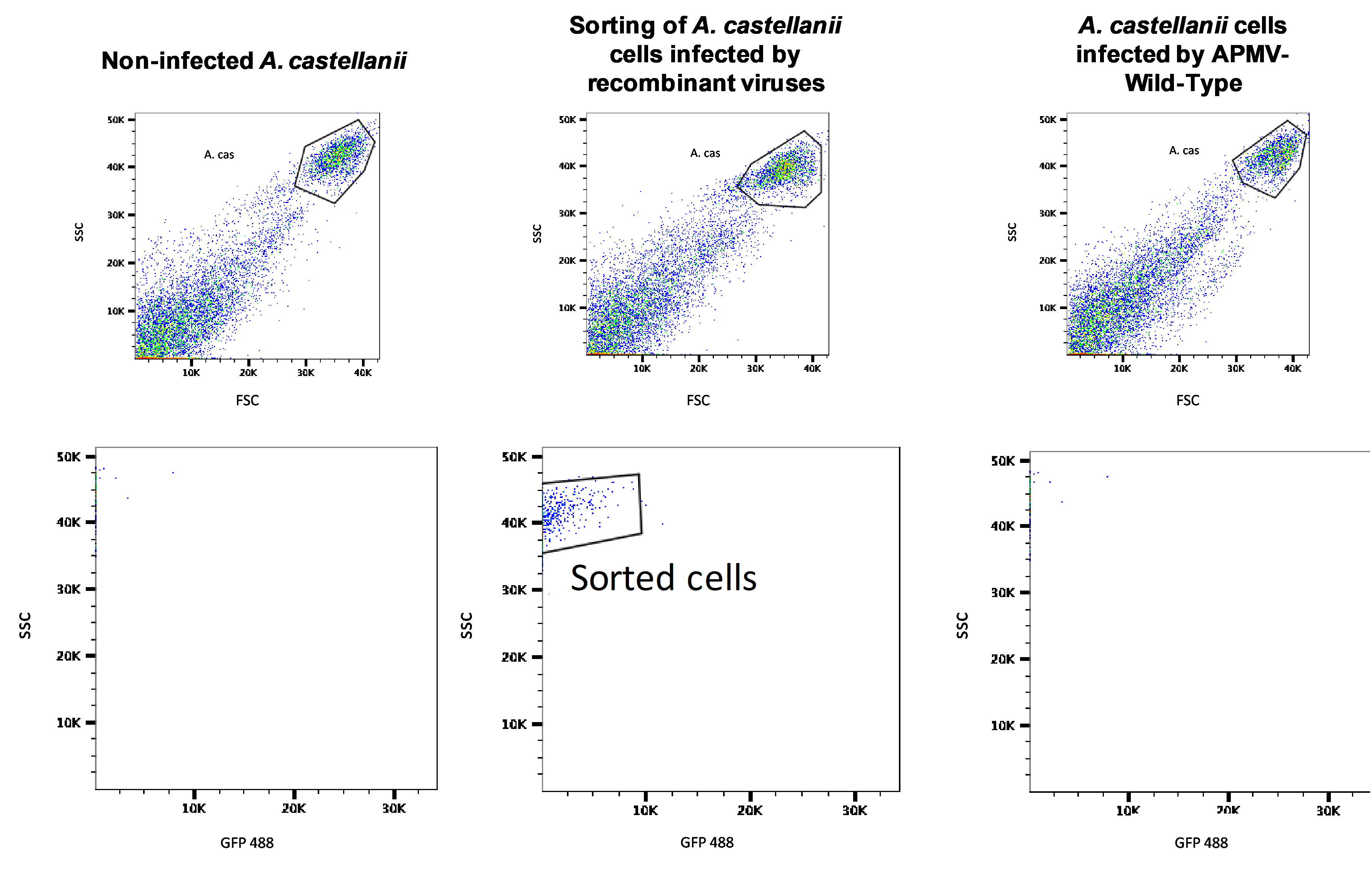

Supplement: FIGURE S1 — APMV R349-KO infected amoebas screening and sorting by BD FACS Scan flow cytometer. The cutoff was defined based on the background fluorescence detected in uninfected and APMV-wild-type infected cells. [file Image_1.TIF]

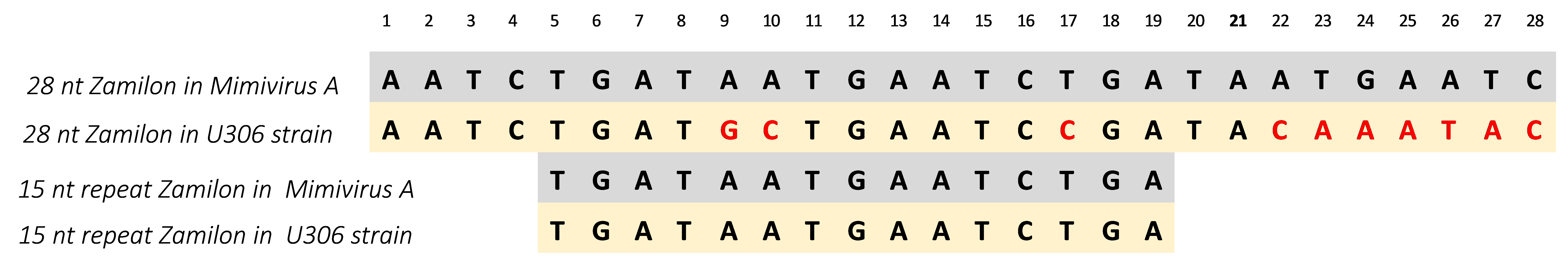

Supplement: FIGURE S2 — Sequence homology representation between different Zamilon sequences present in the MIMIVIRE of APMV and U306 strain. The 28-nucleotides sequence of Zamilon present in the initial R349 gene of mimiviruses A is shortened to a sequence of 21 nucleotides with 3 mismatches (in red) in the ORF 363 of Mimivirus U306 strain. The repeat of 15 nucleotides (albeit single) located in the ORF 364 match perfectly the original 28-nucleotides sequence (and thus the four repeated sequence) found in the other mimiviruses from lineage A. [file Image_2.TIF]
